# Parental and medical knowledge and management of fever in Italian pre-school children

**DOI:** 10.1186/1471-2431-12-97

**Published:** 2012-07-13

**Authors:** Elena Chiappini, Alessandra Parretti, Paolo Becherucci, Monica Pierattelli, Francesca Bonsignori, Luisa Galli, Maurizio de Martino

**Affiliations:** 1 Department of Sciences for Woman and Child’s Health, University of Florence, Florence, Italy; 2 Primary Care Paediatricians, Florence, Italy; 3 Anna Meyer Children’s University Hospital, Viale Pieraccini, 24- I-50139, Florence, Italy

**Keywords:** Fever, Children, Fever phobia, Survey, Paediatrician, Doctor, Caregiver/parent

## Abstract

**Background:**

Guidelines for the management of fever in children have been recently published, however “fever phobia” is still spreading. To provide information which may sustain educational interventions tailored to our population we investigated the parental and medical knowledge and management of fever in preschool children.

**Methods:**

A questionnaire was administered to a convenient sample of Italian parents and paediatricians. The questionnaire elicited information about definition and cause of fever, concerns about fever, method of temperature measurement, and treatment modalities.

**Results:**

Overall, 388 parents and 480 paediatricians were interviewed. All the parents believed that fever could cause at least one harmful effect and 89.9% (n = 349) believed that, if left untreated, it can cause brain damage or seizures. Parents used multiple resources to obtain information about fever but 67.8% (n = 264) considered paediatricians as their primary resource. Several wrong behaviours were found in the same proportions among parents and paediatricians: 78.5% of paediatricians (n = 377) and 77.8% of parents (n = 302) used physical method to reduce fever (P = 0.867); 27.0% of paediatricians (n = 103) and 21.4% (n = 83) of parents declared to alternate ibuprofen and acetaminophen (P = 0.953). Differently, 73.1% (n = 351) of paediatricians preferred oral to rectal administration of antipyretics compared to 48.7% (n = 190) of parents (P < 0.0001). Worrisomely, 1.4% of paediatricians and 1.2% of parents declared to use acetylsalicylic acid or steroids as second-choice antipyretics (P = 0.937) and 6.7% (n = 26) of parents declared to use table- or teaspoons for determining the dose of drug.

**Conclusions:**

Paediatricians’ attitudes greatly influence the parental behaviours and beliefs. Implementation of educational programs regarding the management of the febrile child are needed in our setting.

## Background

In 1980 Dr Schmitt was the first to coin the term “fever phobia” to describe parents’ unrealistic fears about fever associated with numerous misconceptions about its management, and its role in illness. [1] Since then, several reports described the spread of this attitude among parents and paediatricians or nurses in Europe and US [2-12]. However studies which simultaneously assessed parental and paediatric knowledge and management of fever are poor [13,14] We suppose that, at least in our setting, pediatricians’ fear and attitude toward fever play a crucial role in driving the parental fever-phobia. The Italian Health System covers the entire population of the Italian children aged 0-14 years. Every child is assigned to a certain paediatrician since birth and he/she is subsequently followed-up by the same physicians for years. Thus, the family paediatrician has a strategic role, as he/she reaches children belonging to all social classes and parents usually develop full confidence in him/her. In order to provide information which may sustain educational interventions tailored to our population we investigated the parental and paediatricians’ knowledge and management of fever in preschool children.

## Methods

### Survey given to parents and paediatricians

Subjects were interviewed by the use of a questionnaire, developed on the bases of other previous similar surveys [1,4] and on the recent UK and Italian guidelines for the management of the febrile child. [15,16]. For the parental survey, a questionnaire, based on the study of Schmitt *et al*. 1980 and Crocetti *et al*. 2001 [1,4] was developed to elicit information about definition of fever, concerns about fever and fever management. Additional information included methods and frequency of temperature monitoring, used methods for body temperature control, sources of information and beliefs regarding potential consequences of fever [1,4,7,14].

All the parents of children aged 0-6 years attending 12 public nursery-schools, all located in the same municipality (Lastra a Signa, Florence, Italy) were invited to participate in the study by completing questionnaires between March and June 2010. Non-Italian parents were preliminarily asked whether they were able to read and write in Italian. The questionnaire was administered by one paediatrician (investigator A.P.) to the parent accompanying the child at the nursery-school in the morning who also obtained the verbal consent for the study and remained present while the parent was completing the questionnaire. The instrument consisted of 18 questions covering issues common to fever and its management (Appendix). Parents were asked to choose responses from a checklist. Multiple-choice items could be given at questions 1,2,9,10,12,14,15,16. In addition, demographic data were gathered for estimation of socioeconomic status.

For the medical survey, a questionnaire was developed and administered to all the paediatricians attending the 14^th^ National Congress of Practice Paediatrics, held in Florence on November 2009. The content of the questions was similar to parental questions, but the terminology was adapted to the study group. The questionnaire consisted of 16 questions (Appendix) and questions 1,2,3,5,6,7,9,11 are the same of those administered to caregivers/bystanders.

These surveys were approved by the ethics committee of the Anna Meyer Children University Hospital.

### Statistical analysis

Results were given as absolute numbers and percentages. The percentage of responses to the questions has been calculated on the total of participants. Differences in responses between parents and paediatricians were evaluated by contingency table analysis with the *χ*^2^ or the Fisher’s exact test (2 grades of freedom), as appropriate. SPSS software package (SPSS 11.5; Chicago, IL) was used, and p < 0.05 was considered as statistically significant.

## Results

### Results of questionnaire administered to parents

Overall 388/644 (60.2%) parents agreed to be included in the study and completed the survey. Demographic data of the study population and distributions of the responses in the different school are summarized in Table 1. Most of the participants were mothers (86.9%), had an Italian origin (86.1%), were aged 31-40 years, and had a high school diploma or an university degree (56.2%).

**Table 1:** Demographic data of parents participating into the study (n = 388). IQR:Interquartile range

**Variable**	**n (%)**
Gender	
Female	337 (86.9)
Male	51 (13.1)
Age	
20-30	39 (10.1)
31-40	271 (69.8)
41-50	78 (20.1)
Number of children	
1	117 (30.1)
2	232 (59.7)
3 or more	39 (10.0)
Child’s age	
0-3 months	2 (0.5)
4-36 months	95 (24.5)
>36 months	291 (75.0)
Median age of children (months)	
First child	60 (IQR 36-90)
Second child	48 (IQR 24-60)
Country of origin	
Italian	334 (86.1)
European	35 (9.0)
Other country	19 (4.9)
Educational level	
Elementary school	4 (1.0)
Intermediate school	127 (32.7)
High school	180 (46.4)
University	77 (19.8)
Employment status	
Housewife	49 (12.6)
Part time-work	130 (33.5)
Full time-work	161 (41.5)
Distribution of the responders in the schools	
Nursery schools	
I	23 (5.9)
II	30 (7.7)
III	71 (18.2)
IV	45 (11.6)
V	27 (6.9)
VI	14 (3.6)
VII	10 (2.5)
VIII	45 (11.6)
IX	26 (6.7)
X	15 (3.8)
XI	54 (13.9)
XII	28 (7.2)

Results regarding temperature monitoring methods are given in Table 2. All the parents believed that fever could cause at least one harmful effect (Table 3). Most of them chose two or more answers (two answers n = 115; 29.6%, three answers n = 53; 13.6%, four answers n = 13; 3.3%). Parents used multiple resources to obtain information about fever (see Table 3). Paediatricians were their primary resource for this information for many parents and approximately half the parents used the information from the administration instructions with the preparations. No parents reported using information from the media (television, internet) to determine how to manage childhood fever.

**Table 2:** Temperature monitoring method used by parents (n = 388) and paediatricians (n = 480) participating into the study

	Parents n (%)^*^	Paediatricians n (%)*	**P**
Site/Mode of measurement			
Axillary	318 (82.0)	388 (80.8)	0.737
Rectal	62 (16.0)	39 (8.1)	<0.0001
Groin crease	7 (1.8)	32 (6.6)	0.001
Oral	4 (1.0)	0 (0.0)	0.084
Auricular	26 (6.7)	9 (1.8)	0.001
Plastic strip placed on forehead	31 (7.9)	11 (2.2)	<0.0001
Type of thermometer owned/recommended			
Mercury-in-glass	203 (52.3)	108 (22.5)	<0.0001
Digital	255 (65.7)	305 (63.5)	0.551
Auricular	32 (8.2)	10 (2.0)	<0.0001
Skin Infrared	27 (6.9)	5 (1.0)	0.330
Plastic strip placed on forehead	1 (0.2)	0 (0.0)	0.915
Dummy-pacifier style	1 (0.2)	0 (0.0)	0.915
No thermometer owned/recommended	0 (0.0)	52 (10.8)	<0.0001

*total is more than 100% because parents and paediatricians may have given multiple answers.

**Table 3:** Beliefs regarding harmful effects and possible highest degree of fever, intervals of fever monitoring, and resources of information reported by 388 parents

**Harmful effects of fever, reported by parents**	**n (%)**
·Seizure	319 (82.2)*
·Brain damage	30 (7.7)
·Death	15 (3.8)
·Dehydration	174 (44.8)
·Really sick	21 (5.4)
·Coma	10 (2.5)
·Delirium	87 (22.4)
·Blindness	3 (0.7)
·Other	19 (4.9)
Belief that if left untreated fever can reach	
·<40.6 °C	172 (44.3)
·40.7-43.2 °C	204 (52.6)
·> 43.3 °C	12 (3.1)
Time intervals of fever monitoring	
·<16 minutes	19 (4.9)
·16-30 minutes	33 (8.5)
·31-60 minutes	82 (21.1)
·61-120 minutes	130 (33.5)
·>121 minutes	124 (32.0)
Declared source of information	
·According to my paediatrician order	264 (67.8)*
·Reading the package leaflet of medicinal/advice line	188 (48.3)
·Consulting other persons	1 (0.2)
·According to information gathered by internet, TV, papers	0 (0.0)
·According to the dose that paediatricians had advised me previously	46 (11.8)

^*^Total is more than 100% because parents may have given multiple answers.

Physical methods of temperature control were used by 302 (77.8%) parents (Table 3). About 5.0% of parents (n = 19) declared that they would give antipyretics for body temperature < 37.8 °C. Most of parents reported using acetaminophen (n = 375; 96.6%) or ibuprofen (n = 113; 29.1%) to lower the body temperature, but, worrisomely, some parents (n = 2; 0.5%) reported using aspirin and 0.7% (n = 3) other drugs such as steroids or metamizole. Twenty-one percent of parents (n = 83) declared to use usually combined or alternating administration of ibuprofen and acetaminophen.

Awareness of overdose-misuse risk of antipyretics: 6.7% of parents (n = 26) declared to use table- or tea-spoons for determining the dose of drug instead of a standardized measuring device (cup or syringe) for oral solution. Even if rectal administration should be considered only in the presence of vomiting, the 51.0% of parents (n = 198) declared to administer rectal suppositories routinely. A substantial proportion of parents (n = 71; 18.3%;) stated to prefer suppository to oral formulation because it’s easier to administer to the child. Other reported reasons were that suppositories were considered to be more effective or faster in acting (n = 77; 19.8%), and that they had been advised to use suppositories by their paediatrician (n = 44; 11.3%). Only 30.9% (n = 129) of parents used rectal suppositories because of the impossibility to administer the drug orally (i.e. because of vomiting).

### Results of questionnaire administered to paediatricians

Among 648 paediatricians attending the National Congress of Practice Paediatrics, held in Florence on November 2009, 480 (74.0%) returned the questionnaire. No demographic data were collected from paediatricians (Appendix).

Sixty-four percent of paediatricians (n = 309) believed that body temperature should be measured rectally in children aged < 1 year while only 23.1% (n = 111) would measure it axillary. In children aged >1 year, 80.8% (n = 388) believed that axillary measurement should be used (Table 1). The favourite type of thermometer was the digital thermometer (n = 305; 63.5%). A tympanic measurement using an infrared thermometer was recommended in the hospital care setting by 48.3% (n = 232) of the paediatricians. Only 7.0% (n = 34) declared that it could be used also by parents at home, while 174/480 (36.2%) would use it in both these situations.

The temperature that paediatricians would regard as fever was above 37.0 °C for 14.3% (n = 69), 37.5 °C for 32.7% (n = 157), 38.0 °C for 41.2% (n = 198). Sixty-nine percent of paediatricians (n = 335) declared that they would give antipyretics for temperatures >38.5 °C, 85/480 (17.7%) above 38.0 °C, and 56/480 (11.6%) above 39.0 °C.

Sixty-five percent (n = 315) of paediatricians declared to recommend physical methods, such as sponging or ice pack, to reduce fever only if the temperature is not going down after the antipyretic drug. Thirteen percent (n = 62) declare to suggest the use physical methods in association with antipyretic drugs. Paracetamol was the first choice antipyretic drug for 96.4% (n = 463) of paediatricians and ibruprofen was the second choice antipyretic drug for 91.6% of them (n = 440). No paediatricians declared to use of acetylsalicylic acid or steroids as first choice, but, worryingly, 7/480 (1.4%) of them declared to use them as possible second choice drugs.

#### Awareness of overdose-misuse risk of antipyretics

Correctly, oral administration of paracetamol was preferred to rectal administration by 73.1% of paediatricians (n = 351) and rectal administration was considered only in the presence of vomiting by 56.2% (n = 270) of paediatricians. However, 24.3% (n = 117) of paediatricians declared to prefer rectal administration because it seems to be more practical. The half (n = 240; 50.0%) of the paediatricians declared to use a higher pro-kilo dosage of paracetamol when it is administered rectally. Most of them (n = 273; 56.8%) used to give information about fever management at the first vaccinations, with a written prescription (n = 294; 61.2%). Alternate use of ibuprofen and paracetamol was adopted by 130/480 (27.0%) of paediatricians. Contrary to the guidelines recommendations, preventive use of paracetamol or ibuprofen was recommended for the prevention of febrile convulsion in febrile children by 60.6% (n = 291) of paediatricians.

### Comparison between parents’ and paediatrician’s answers

**Figure 1:**
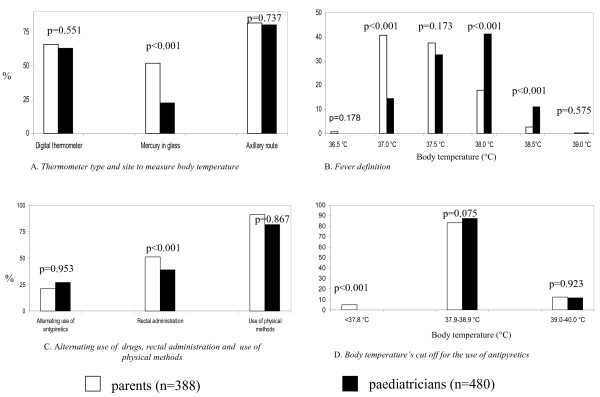
Comparison between parents’ and paediatricians’ answers.

The temperature parents reported representing fever was inversely related to the paediatricians’ reports, which more closely reflect the current evidence (15,16). Alternately, both parents and paediatricians had similar temperatures for administering antipyretics (Figure 1).

There were not substantial differences regarding the kind of thermometer used (being the digital thermometer the most used/recommended), the site to measure body temperature (being the axillary site the most frequently used/recommended), the kind of antipyretic drug and values of body temperature reported to be considered as a cut off to treat fever (Table 2 and Figure 1). Some wrong behaviours were observed in similar proportions in parents and paediatricians. In spite of guidelines recommendations, 78.5% of paediatricians (n = 377) and 77.8% of parents (n = 302) used physical method to reduce fever (P = 0.867); 27.0% of paediatricians (n = 103) and 21.4% parents (n = 83) used the combined or alternating use of ibuprofen and acetaminophen (P = 0.953) (Figure 1). Worrisomely, 1.4% of paediatricians and 1.2% of parents declared to use acetylsalicylic acid or steroids as second-choice antipyretics (P = 0.937). On the other hand, 73.1% (n = 351) of paediatricians, correctly, preferred oral administration of antipyretics compared to 48.7% (n = 190) of parents (p < 0.0001) (Figure. 1). More parents than paediatricians (n = 198; 51.0% [n = 198] vs. 24.3% [n = 117]; P < 0.0001)) declared to use/recommend the suppositories because they think that they are more practical than oral formulation and, in general 43.75% (n = 210) of paediatricians and 70.5% (n = 120) of parents declared to use suppositories for reasons other than vomiting (P < 0.0001) (Figure 1).

## Discussion

The present study is an analysis of current beliefs about fever and behaviours in 388 parents and 488 Italian paediatricians. Results were analyzed considering guidelines recommendations [15,16]. Alarmingly, there was poor awareness about the real risk of misuse of antipyretics. One third of parents thought that an higher dose of antipyretics is not dangerous, but it’s not useful and the half of pediatricians used a higher dose of paracetamol when it is given rectally. One major finding is that similarities between paediatricians’ and parents’ practices have been observed, including use of physical methods to reduce body temperature, use acetylsalicylic acid or steroids as possible alternative antipyretics, combined or alternating use of ibuprofen and acetaminophen, spread use of rectal acetaminophen, and use of antipyretics with the aim to prevent febrile convulsions, despite the fact that all these practices are discouraged by the current guidelines. [15,16]. This is consistent with the fact that the majority of parents reported to consider their paediatrician as their primary resource for information about fever. This is different to what is reported by other authors in the U.S., Israel or Canada, describing a higher proportion of parents declaring to obtain information regarding the management of fever from friends, or media [1,4,14,17]. This finding may be due to the peculiar organization of the Italian Health System: all the children are assigned to a certain paediatrician since birth, without any charge to pay for medical visits, and they will be followed very closely until 14 years of age.

Other authors previously investigated knowledge and behaviours of paediatricians or nurses [12,14,18-23]. Similarly to our results, dangerous practices as the use of alternating antipyretics, rectal administration of drugs, or the use of antipyretics for the prevention of febrile convulsions, were found to be recommended by a large share of paediatricians [23]. These results are particularly alarming because, even if antipyretic drugs are largely demonstrated to be safe and effective [15,16], it is reported the possibility of their toxic effects, if not correctly used [15,16]. Few previous studies simultaneously reported data from parents and paediatricians/nurses [13,14].

In the early 2000’s Sarrell *et al.*[13] conducted a large survey among paediatricians, nurses and parents in Israel. Several discrepancies were observed between parents and paediatricians/nurses habits. For example, the majority of parents believed it necessary to treat children with low-grade fever without any other sign of illness, whereas the physicians and nurses did not [13]. Differently, in our study, despite the fact that the body temperature that parents reported representing fever was inversely related to the paediatricians’ reports, both parents and paediatricians used similar cut-offs for administering antipyretics. A similar survey was also conducted by Karwoskwa *et al.* in Canada in 2002 [14]. The temperature cut offs considered to be fever by parents in that study were similar to those that we observed and, in the same way, most of the parents declared to be concerned about discomfort, seizures and dehydration associated with fever [14]. Taken together these results suggest that adoption of guideline recommendations is not improving over years and it is similar among different countries. We noticed that rectal administration of paracetamol has been frequently reported in the US but less commonly in the UK or Australia [15,16], suggesting that adoption of some behaviours is more influenced by local cultural attitudes than by scientific evidence.

Our findings underline the importance of educational interventions in paediatricians in order to modify, consequently, the parents’ behaviours and to improve their knowledge about fever. Paediatricians have an unique opportunity to make an impact on parental understanding of fever and its management. Future studies are needed to evaluate the effectiveness of such interventions.

### Study Limitations

Our investigation has potential limitations. Our results may not generalize to all paediatricians. Paediatricians included in the study constituted approximately 6.0% of all the about 7500 Italian paediatricians currently working in Italy and were all attending an annual National Congress of Practice Paediatrics on November 2009. Therefore, our study population may be not representative of all Italian paediatricians. Data regarding residence of paediatricians were not collected. Thus, our study does not provide information regarding possible differences in responses according to the geographical provenience The parents sample (parents of children attending 12 nursery-schools in Florence) may be not representative of the entire population. Preliminary, parents were required whether they could read and write in Italian, but we can not exclude that non-Italian parents may had encountered any comprehension problem. It is well known that self-reported behaviours can be misleading since some participants might not complete the survey as carefully as they would act in real settings. [24]. Finally, participants, both parents and paediatricians, could be more interested in fever management than those who did not agree to participate into the study.

## Conclusions

“Fever phobia” remains extremely widespread among parents and the vast majority believes that fever is harmful. Parents consider paediatricians as their primary source of information and this is demonstrated also by the consistency between the responses in the two groups. Some of identified behaviours (widespread use of suppositories, alternating use of antipyretics, use of spoons and teaspoons to dose antipyretics) expose children to the risk of overdose. Educational programs targeted to educate paediatricians may be an effective action to change the parents’ understanding and management of fever.

## Appendix 1 Questionnaire for parents

### Demographic data

Kinship: a)mother b)father c)other

How old are you?

How many children do you have?

How old are your children?

Where are you from?

What is your qualification? a) elementary school b) intermediate school c) high school d) university

What is your qualification? a) elementary school b) intermediate school c) high school d) university

### Questionnaire

1. When you take the temperature of your child which is the best site?

a) the armpit

b) the rectum

c) groin crease

d) the mouth

e) the ear

f) on the forehead

2. What kind of thermometer do you use to measure your child’s temperature?

a) mercury-in-glass

b) electronic

c) auricolar

d) skin infrared

e) plastic strip placed on forehead

f) “dummy”

g) e) I don’t have a thermometer

3. Which the cut off of body temperature that you consider to define fever?

a) 36.5 °C

b) 37 °C

c) 37.5 °C

d) 38 °C

e) 38.5 °C

f) 39 °C

4. Which is the body temperature cut-off that you consider for high fever?

a) <37.8 °C

b) 37.9-38-9 °C

c) 39-40 °C

d) >40 °C

5. When do you administer antipyretics (body temperature cut off)?

a) <37.8 °C

b) 37.9-38-9 °C

c) 39-40 °C

d) >40 °C

6. If your child had fever and you didn’t treat it, how high could it go?

a) <40.6 °C

b) 40.7-43.2 °C

c) >43.3 °C

7. If your child has fever, how often do you continue to take his temperature?

a) <15’

b) 16’-30’

c) 31’-60’

d) 61’-120’

e) >120’

8. What side effects may a fever cause?

a) seizure

b) brain damage

c) death

d) dehydration

e) really sick

f) coma

g) delirium

h) blindness

i) other

9. Which antipyretic drugs do you administer ?

a) acetaminophene

b) ibuprofen

c) aspirin

d) other (metamizole,betamethasone)

10. When the temperature is not going down, do you believe it is useful to associate two or more antipyretic drugs?

a) yes

b) no

11. Which other remedies for body temperature control do you use in addition to antipyretic drugs to reduce fever in your child?

a) cold sponging

b) ice pack

c) tepid sponging

d) other

e) I use only antipyretic drug

12. How do you administer antipyretic drug?

a) orally

b) rectally

13. If so, why do you administer antipyretic drug rectally?

a) it’s more usefull

b) it’s more practical

c) because doctor said to me

d) if I am not able to give it orally because of child’s refusal

e) if I am not able to give it orally because of vomit

14. How do you calculate the right dose of antipyretic drugs to administered to your child?

a) according to my paediatrician’s order

b) reading the package leaflet of medicinal/advice line

c) consulting other persons

d) according to information gathered by internet, TV, papers

e) according to the dose that my paediatrician had advised me previously

15. To administered antipyretic drug to your child do you consider?

a) the weight

b) the height

16. What do you think about a largest dose of antipyretic drug during an high fever?

a) it’s more efficacious

b) it’s more dangerous

c) it’s not dangerous, but it’s efficacious

17. Which instrument do you use to determine the right dose of antipyretic drug?

a) tablespoons or teaspoons

b) specific dosimeter of the antipyretic drug

c) dosimeters of other drugs

## Appendix 2

### Questionnaire for paediatricians

1. Where should body temperature be measured in children under one year?

a) the armpit

b) the rectum

c) groin crease

d) the mouth

e) the ear

f) on the forehead

2. Where should the body temperature be measured in children over one year?

a) the armpit

b) the rectum

c) groin crease

d) the mouth

e) the ear

f) on the forehead

3. What kind of thermometer do you suggest to measure temperature?

a) mercury-in-glass

b) electronic

c) auricolar

d) d)skin infrared

e) plastic strip placed on forehead

f) “dummy”

g) I don’t suggest any particular thermometer

4. Infrared thermometer must be used:

a) by skilled labours in the hospital/ambulatory setting

b) by parents at home

c) in both situations

5. Over what temperature do you consider that a child has fever?

a) 36.5 °C

b) 37 °C

c) 37.5 °C

d) 38 °C

e) 38.5 °C

f) 39 °C

6. Above what temperature do you administer antipyretics?

a) <37.8 °C

b) 37.9-38-9 °C

c) 39-40 °C

d) >40 °C

7. Which antipyretic drugs do you usually suggest to use?

a) acetaminophene

b) ibuprofen

c) aspirin

d) other (metamizole,betamethasone)

8. Which other drugs do you suggest in addition to the previous?

a) acetaminophene

b) ibuprofen

c) aspirin

d) other (metamizole,betamethasone)

9. When the temperature is not going down quickly, do you believe it is useful to associate two or more antipyretic drugs?

a) yes

b) no

10. Do you suggest to use physical methods as sponging or ice pack to reduce a child’s body temperature?

a) yes, with the antipyretic drug

b) yes, before the antipyretic drug

c) only if the temperature is not going down after the antipyretic drug

d) no, never

11. How do you suggest to administer antipyretic drug?

a) orally

b) rectally

12. If so, why do you suggest to administer antipyretic drug rectally?

a) it’s more usefull

b) it’s more practical

c) because parents prefer this way

d) only in the presence of vomiting

13. Do you suggest a higher dose of antipyretic drug when you administer it rectally?

a) yes

b) no

14. When do you give preventive information about fever management?

a) at the first medical examination of the newborn

b) at the first vaccinations

c) six months’ examination

d) one year’s examination

e) I don’t give any preventive information about fever management

15. Do you give a written prescription regarding modes and administration of antipyretic drugs?

a) yes

b) no

c) often

d) rarely

e) only for patients who have difficulty in comprehension

16. Do you think that antipyretics should be used to prevent febrile convulsions in children?

a) yes

b) no

## Competing interests

The authors declare that they have no competing interests.

## Authors’ contributions

EC participated in the design of the study and performed the statistical analysis and wrote the draft. AP administered the questionnaires, took the verbal consent and wrote the draft. PB and MP participated in the collection and analysis of paediatricians questionnaires. LG and MdM read and approved the final manuscript. All authors read and approved the final manuscript.

## Pre-publication history

The pre-publication history for this paper can be accessed here:

http://www.biomedcentral.com/1471-2431/12/97/prepub
